# Development of a quaternary ammonium poly (amidoamine) dendrimer-based drug carrier for the solubility enhancement and sustained release of furosemide

**DOI:** 10.3389/fchem.2023.1123775

**Published:** 2023-02-17

**Authors:** E. Murugan, V. Yogaraj

**Affiliations:** Department of Physical Chemistry, School of Chemical Sciences, University of Madras, Guindy Campus, Chennai, Tamil Nadu, India

**Keywords:** PAMAM dendrimer, congestive heart failure, solubility enhancement, sustained release, biocompatibility

## Abstract

Furosemide (FRSD) is a loop diuretic that has been categorized as a class IV drug according to the Biopharmaceutics Classification System (BCS). It is used in the treatment of congestive heart failure and edema. Owing to low solubility and permeability, its oral bioavailability is very poor. In this study, two types of poly (amidoamine) dendrimer-based drug carriers (generation G2 and G3) were synthesized to increase the bioavailability of FRSD through solubility enhancement and sustained release. The developed dendrimers enhanced the solubility of FRSD 58- and 109-fold, respectively, compared with pure FRSD. *In vitro* studies demonstrated that the maximum time taken to release 95% of the drug from G2 and G3 was 420–510 min, respectively, whereas for pure FRSD the maximum time was only 90 min. Such a delayed release is strong evidence for sustained drug release. Cytotoxicity studies using Vero and HBL 100 cell lines through an MTT assay revealed increased cell viability, indicating reduced cytotoxicity and improved bioavailability. Therefore, the present dendrimer-based drug carriers are proven to be prominent, benign, biocompatible, and efficient for poorly soluble drugs, such as FRSD. Therefore, they could be convenient choices for real-time applications of drug delivery.

## Introduction

Over the past 2 decades, the poor aqueous solubility among new drug candidates is periodically acknowledged, and it is considered one of the biggest challenges for pharma industries (Van den Mooter, 2011; Baird and Taylor, 2012; Lipinski et al., 2012). The classification of drugs based on the properties of aqueous solubility and intestinal permeability has been described by Biopharmaceutics Classification System (BCS) (Amidon et al., 1995; Yu et al., 2002). The statistical data reveal that 40% of new chemical entities (NCEs) from pharmaceutical industries are identified as hydrophobic compounds with poor aqueous solubility (Sakthivel and Florence, 2003; Pushkar et al., 2006). In general, the degree of therapeutic efficiency of any drug usually depends on the extent of solubility. Additionally, it is known that the aqueous solubility of a drug is a key property as it governs dissolution, absorption, and efficacy *in vivo*. In general, a drug administered in solution form is immediately available for absorption (Vogtle et al., 1978; Tomalia et al., 1985). Multicomponent crystals have been developed in the pharmaceutical field because of their active pharmaceutical ingredients (APIs) (Duggirala et al., 2016; Berry and Steed, 2017). Both cocrystals and salts have been shown to improve many manufacturing and biopharmaceutical properties within APIs. Additionally, it is necessary to mention that the development of any method that helps to promote the solubility of poorly soluble drugs is a revered area of current research. This kind of research certainly supports the pharma industries and technology as its outcome can lead to an increase in bioavailability (Yiyun et al., 2008).

It is well known that furosemide (FRSD) is a loop diuretic drug (4-chloro-N-furfuryl-5-sulphamoylanthranilic acid) used for the oral treatment of edema with cardiac, renal, and hepatic failure, as well as hypertension (Rowbotham et al., 1976). As per the BCS classification system, FRSD is classified as a class IV drug (i.e., low solubility and low permeability) and thus has low oral bioavailability and solubility. It is absorbed rapidly from the gastrointestinal (GI) tract with a half-life of 30–120 min. Its bioavailability has been reported to be approximately 60%–70%, but its absorption is variable and erratic (Granero et al., 2010). Additionally, to alleviate the poor aqueous solubility of FRSD, many methods have been adopted, including salt formation, (Berge et al., 1977), prodrug formation, (Rautio et al., 2008), particle size reduction, (Rabinow BE, 2004), complexation, (Loftsson and Duchene, 2007), and the use of micelles, (Lavasanifar et al., 2002), microemulsions, (Mueller et al., 1994), nanoemulsions, (Fatouros et al., 2007), nanosuspensions of FRSD, (Müller and Peters, 1998), and solid-lipid nanoparticles, (Potta et al., 2010), as well as ‘mixed solvency’ using organic solvents. Unfortunately, none of these methods completely resolved the issues, and all of them have their own merits and demerits.

However, recently, the development of dendrimer-based drug carriers has attracted greater attention in the field of biomedical application (Yoo and Juliano, 2000; Choi et al., 2004). Dendrimers are highly branched and reactive three-dimensional macromolecules, with all bonds emanating from a central core. Since their introduction in the mid-1980s, these novel classes of polymeric materials have attracted considerable attention due to their unique structure and properties. More specifically, compared with traditional linear polymers, dendrimers have much more accurately controlled structures, with a globular shape, and tailor-made peripheral functionalities. Particularly, as a novel class of polymer materials with unique architectures, PAMAM dendrimers have been studied in a wide range of applications, including drug delivery, (Esfand and Tomalia, 2001; Patri et al., 2002), gene therapy, (Kukowska-Latallo et al., 1996; Eichman et al., 2000; Luo et al., 2002), catalysis, (Cheng et al., 2001; Murugan and Vimala, 2013; Murugan and Jebaranjitham, 2015; Murugan and Pakrudheen, 2015), biosensing, (Nevins Buchanan et al., 2004; Shen and Hu, 2004; Snejdarkova et al., 2004), photonics, (Ispasoiu et al., 2000; Senarath-Yapa and Saavedra, 2001; Grabchev et al., 2003), electronics (Grabchev et al., 2002), and environmental remediation (Diallo et al., 1999; Diallo et al., 2004). Hence, drug delivery systems derived from dendrimers offer a method for drug loading and release, which expands pharmacokinetic and pharmacodynamic behaviors. In particular, modified dendrimers have been used as carriers for plasmid DNA, antisense oligonucleotide, and siRNA (Malik et al., 2000; Kobayashi et al., 2003; Kobayashi et al., 2004; Kang et al., 2005; Wada et al., 2005). Moreover, PAMAM dendrimers are being investigated not only for gene therapy but also for drug delivery and bioimaging purposes (Hu C. M. et al., 2010). Devarkonda et al. (2007) used PAMAM dendrimer G3 with amine terminal G2.5 with an ester terminal for solubility enhancement and the slow release of FRSD. These amine terminal groups are toxic and have the drawback of non-biocompatibility in drug delivery. In addition, cell death was also reported by Kuo et al. (2005) It is important to mention that solubility and toxicity issues are always associated with these technologies and hence they were a matter of concern until now; the dendrimer surface has now been neutralized through hydroxylation, PEGylation, acetylation, and glycosylation to reduce cytotoxicity (Majoros et al., 2005; Kolhatkar et al., 2007).

Based on the above reports, PAMAM G2 and G3 dendrimers were neutralized in this study through surface hydroxylation followed by internal quaternization using hydroxylating agent 2-(2-chloroethoxy) ethanol. The modified dendrimers QPAMAM-CH_2_CH_2_OCH_2_CH_2_OH (G2)/(G3) were characterized and demonstrated as drug carriers using a poorly soluble drug, FRSD, as a model for solubility enhancement and sustained release. Based on the favorable results, the present study could be applied to FRSD in pharmaceutical therapy, as well as other poorly soluble drugs.

## Materials and methods

### Materials

PAMAM dendrimers with an ethylene diamine core (EDA) of G2 and G3, 2-(2-chloroethoxy) ethanol, Triethyl amine, methyl iodide, N, N- dimethyl formamide (DMF), and MTT (3-(4, 5-dimethylthiazol-2-yl)-2, 5-diphenyltetrazolium bromide) were procured from Sigma-Aldrich, India. Amberlite IRA-402 Cl resin was obtained from SD Fine Chem. The Float-A-Lyzer and membrane filter were purchased from Spectra Pore and Himedia, respectively. For solubility and *in vitro* release and cytotoxicity studies, PBS (pH 7.4) and GD water were used. FRSD was obtained as a gift sample from Chennai Drug House, Chennai, India. The Vero and HBL 100 cell lines were obtained from NCCS, Pune, India and preserved in the Hubert Envirocare Laboratory, Chennai, India.

### Instrumentation

UV-visible spectra were measured using a PerkinElmer Lambda 35 spectrophotometer with uv-winlab software. Sample analyses were carried out in the wavelength range 200–800 nm under ambient conditions. The Fourier transform infrared (FTIR) spectra and ^1^H and ^13^C NMR spectra were obtained using a Bruker Tensor-27 FTIR spectrometer and a Bruker 500 MHz NMR spectrometer. The MALDI TOF-TOF analyzer from Applied Biosystems 4,800 was used for MALDI-TOF analysis. All mass spectra were obtained by keeping the average of 400 shots with positive ion mode and in reflection mode, and the matrix used was 2, 5-dihydroxybenzoic acid (DHB). PerkinElmer DSC model seven was used for recording DSC thermograms. The particle size and the zeta potential measurements were obtained using a Zetatrac instrument (Microtrac Inc, York, PA, United States). The cytotoxicity study was carried out by Shasam Biologicals Pvt Ltd, Chennai, India.

### Synthesis of two types of quaternary ammonium Poly (Amidoamine) dendrimer drug carriers using 2-(2-chloroethoxy) ethanol as a hydroxylating agent

**SCHEME 1:**
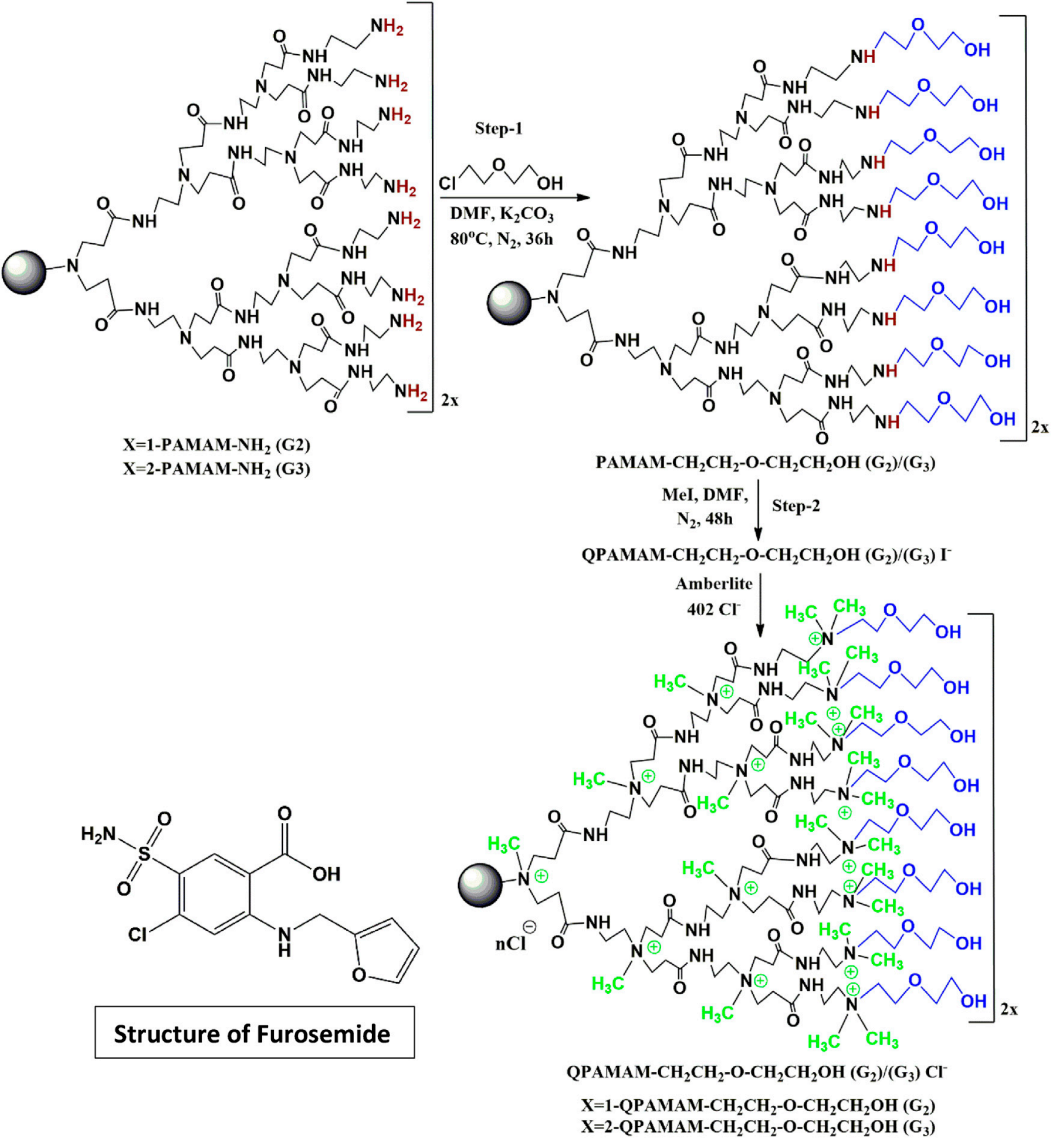
Synthesis of two types of quaternary ammonium dendrimer drug carrier using 2-(2-chloroethoxy) ethanol and the structure of furosemide.

The synthesis was performed in two steps by adopting a procedure published previously with mild modification (Murugan et al., 2014; Murugan et al., 2015). The surface amine groups present in PAMAM (G2) with a molecular weight of 3,256 g/mol and PAMAM (G3) with a molecular weight of 6,909 g/mol were neutralized by ether-linked hydroxyl group 2-(2-chloroethoxy) ethanol (Scheme 1), and quaternary ammonium ions were generated in the internal tertiary amines using methyl iodide. The excess DMF impurities were removed using a vacuum oven to obtain semisolid poly (amidoamine) ethoxy ethanol dendrimer products QPAMAM-CH2CH2OCH2 CH2OH (G2) and QPAMAM-CH2CH2OCH2CH2OH (G3). Finally, for ion exchange, Amberlite anion resin was used, as described in our previous work (Murugan et al., 2013; Yogaraj et al., 2020; Murugan et al., 2022), and the solution was lyophilized for surface hydroxylated and internally quaternized poly (amidoamine) 2-ethoxy ethanol dendrimers with Cl-as the counter ion.

### Solubility study of FRSD

**FIGURE 1:**
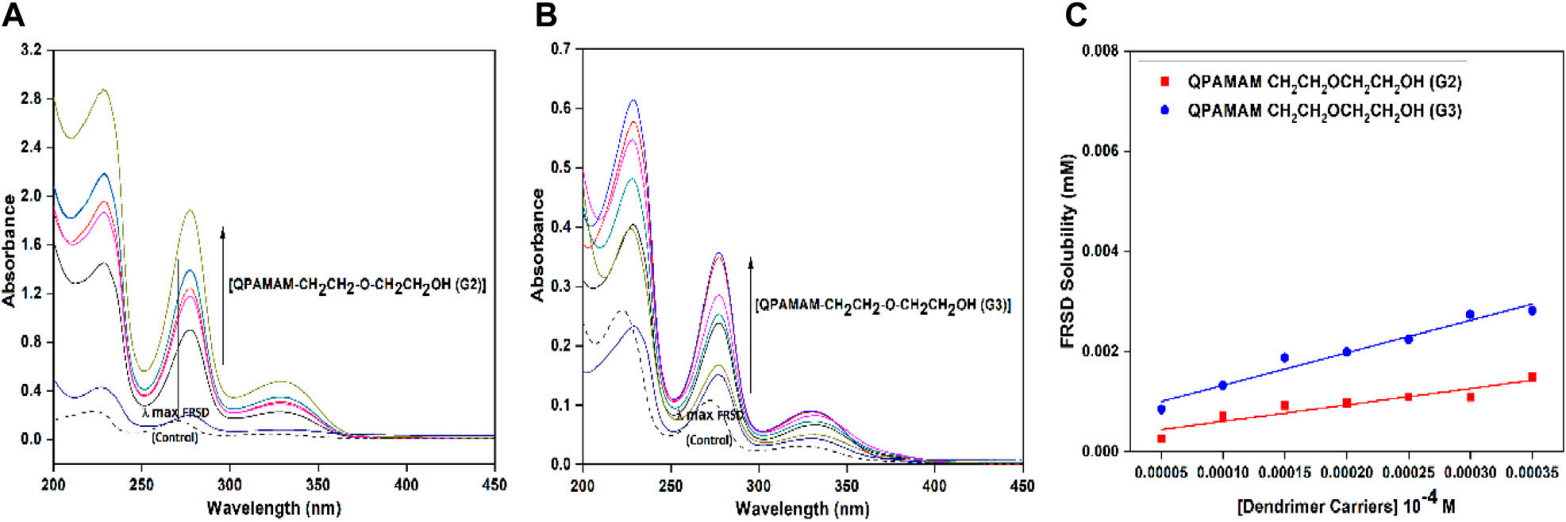
UV-Vis spectra of FRSD (dotted line) and FRSD with different concentrations of drug carrier **(A)** QPAMAM-CH_2_CH_2_OCH_2_CH_2_OH (G2), **(B)** QPAMAM-CH_2_CH_2_OCH_2_CH_2_OH (G3) and **(C)** Solubility behavior of FRSD at various concentrations of two types of quaternary ammonium dendrimer drug carriers in aqueous solutions.

The solubilizing potential of the newly developed drug carriers was evaluated by employing the poorly soluble drug FRSD, and solubility studies were carried out using a UV-visible spectrophotometer. Initially, the calibration curve for the drug was constructed within the specified and valid range of Beer’s law, and the unknown concentration of drug solubilized at a particular concentration of quaternized dendrimer drug carrier solution was estimated. From the stock solution, serial dilutions were made using PBS (pH 7.4) for different concentrations of standard solutions of FRSD. In the UV region, λ_max_ for FRSD in PBS (pH 7.4) was observed at 271 nm (Figures 1A–C) and subsequently at this λ_max_, the amount of FRSD solubilized in the respective standard solutions was estimated. The linearity of the plot was ensured by equation y = (12,709 ± 0.173) x (*R* ^2^ = 0.998) and the absorption coefficient of FRSD is *ε*_max_ = 12,709 M^-1^ cm^-1^. The tentative drug molecules bound per molecule of dendrimeric unit were calculated using the directional coefficients of each drug carriers. The phase solubility technique (Higuchi and Conners, 1965) was employed for the solubility of FRSD in the presence of drug carrier solutions with slight modifications. From the drug carriers, a series of concentrations of solutions from 0.5 × 10^−4^ M to 3.5 × 10^−4^ M were prepared, and an excess of the drug was added to each concentration. Then, the respective vials were sonicated for 30 min and mechanically shook for 24 h at 35 °C. The control experiment for the drug without dendrimer carriers was performed simultaneously. After equilibrium, the dendrimer-drug solutions were centrifuged at 10,000 rpm and filtered to remove undissolved drugs, followed by UV-visible spectrophotometry analysis. UV-vis analysis was repeated three times to confirm the solubility measurements. The extent of drug solubility is indicated by the increase in absorbance, and a calibration curve of FRSD was used to obtain the amount of drug solubilized in the quaternary ammonium dendrimer carrier solution. The blank was also carried out identically to nullify the absorbance due to their respective carrier molecules. From the results, the solubility increments and dose: solubility ratio was calculated and given in Table 1. Additionally, the maximum loading efficiency of the drug was calculated using the formula:

$$Loading\;efficiency\;\left(\%\right)=\frac{Actual\;drug\;content\;\left(AC\right)}{Theoretical\;drug\;content\;\left(TC\right)}$$

AC−Actual quantity of drug present in the carrier

TC−100% theoretical quantity of drug present in the carrier.

**TABLE 1:** Solubility and dose: solubility ratio of FRSD drug in the presence of drug carriers.

[QPAMAM-CH_2_CH_2_OCH_2_CH_2_OH (G2)/(G3)]×10^4^M	Solubility of FRSD in QPAMAM- CH_2_CH_2_OCH_2_CH_2_OH (G2)		Solubility of FRSD in QPAMAM- CH_2_CH_2_OCH_2_CH_2_OH (G3)		Solubility increment		Dose (mg): Solubility ratio (mg/mL) e)^§^	
	×10^3^M	mg/ml (a)	×10^3^M	mg/ml (b)	(a/c^a^)	(b/c^a^)	(d^b^/a)	(d^b^/b)
0.5	4.967	1.642	7.065	2.336	22.47	31.96	24.36	17.12
1.0	5.178	1.712	8.694	2.875	23.42	39.33	23.36	13.91
1.5	6.057	2.000	12.00	3.969	27.40	54.29	20.00	10.07
2.0	7.142	2.362	15.20	5.027	32.31	68.77	16.93	7.96
2.5	9.900	3.274	18.70	6.185	44.79	84.61	12.21	6.47
3.0	11.50	3.803	23.70	7.838	52.03	107.23	10.51	5.13
3.5	12.80	4.233	24.20	8.004	57.91	109.49	9.45	4.99
Dose (mg): intrinsic solubility (mg/mL) (d^#^/c^a^) 547.19								

^a^ c) Intrinsic solubility (S_0_) of FRSD, in water at 25 °C is 0.0731 mg/mL.

^b^ d) Maximum dose recommended by WHO, for FRSD, is 40 mg.

^c^ e) Dose: solubility ratio ≤250, indicating high solubility related to dose (in bold numbers).

### Interaction studies of drug carrier-drug ComplexS

#### UV-vis spectral studies

The nature of dendrimer drug carrier-drug complex QPAMAM-CH_2_CH_2_OCH_2_CH_2_OH (G2/G3)-FRSD was analyzed using a UV-vis spectrophotometer. Shifts in UV absorption maxima were indicative of complex formation. The solutions prepared for solubility studies were diluted to a suitable concentration and then analyzed for interaction. Meanwhile the dendrimers and hyperbranched polymers showed weak or no absorbance in this wavelength; the absorbance obtained from the dendrimer-drug complex solution should be solely due to the drug (Kolhe et al., 2003). The solubility of FRSD in the dendrimer carrier solutions was associated with its characteristic wavelength. The λ_max_ obtained at 271 nm for FRSD was taken as a reference and compared with the λ_max_ values of the complex solutions derived individually with concentration ranges from 0.05 mM to 0.35 mM. The shift in characteristic absorbance (λ_max_) observed between FRSD (control) and the respective dendrimer-drug complex was used to explain the nature of dendrimer-drug interaction. The UV-vis spectra for FRSD correlated with the complex at different concentrations of quaternary ammonium dendrimer drug carriers is shown in Figures 1A, B.

#### Differential scanning calorimetry (DSC) analysis

**FIGURE 2:**
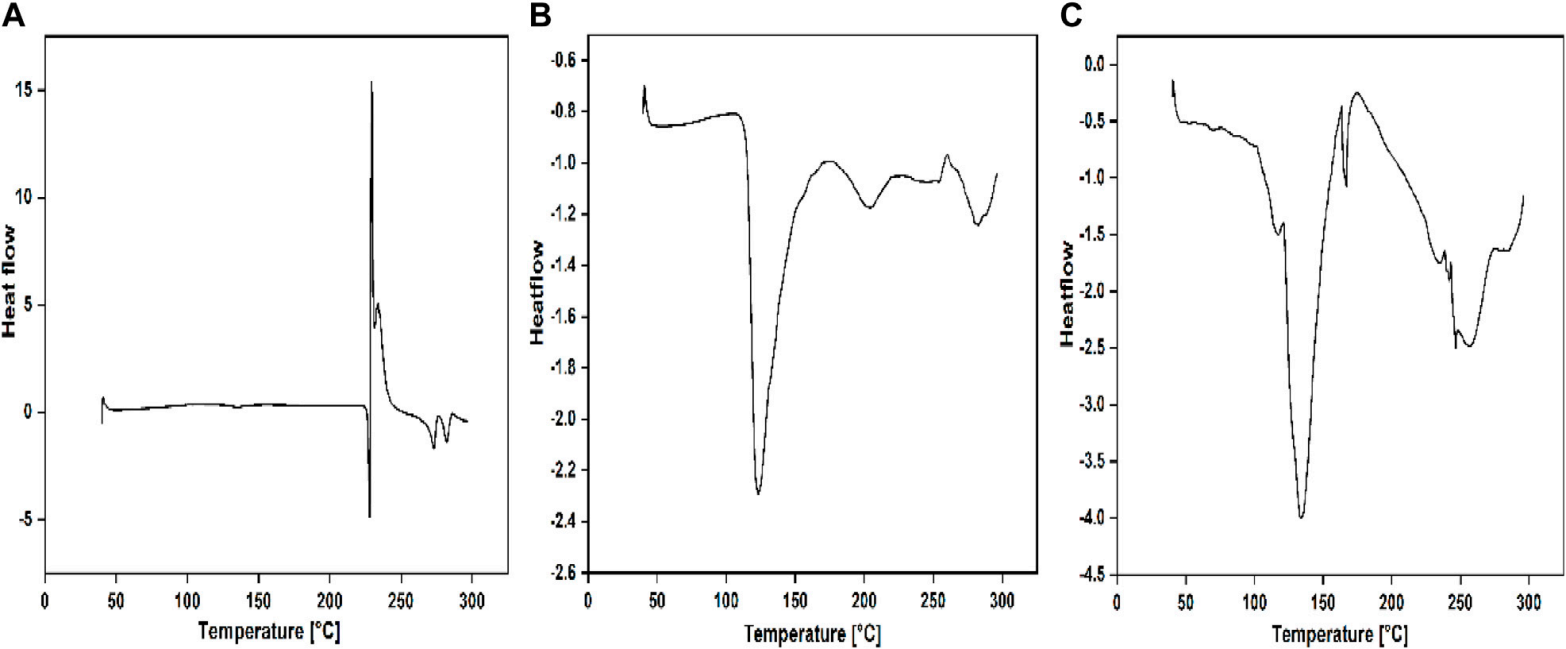
DSC thermograms of **(A)** PAMAM (G3), **(B)** QPAMAM-CH_2_CH_2_OCH_2_CH_2_OH (G3) and **(C)** QPAMAM-CH_2_CH_2_OCH_2_CH_2_OH (G3)-FRSD complex.

The nature of the complex formed between dendrimer drug carriers and FRSD was studied through DSC analysis. The selected complex solution with a higher generation of quaternary ammonium dendrimer drug carrier, i.e., QPAMAM-CH_2_CH_2_OCH_2_CH_2_OH (G3) and its complex QPAMAM-CH_2_CH_2_OCH_2_CH_2_OH (G3)-FRSD, was lyophilized and analyzed for DSC. For comparative purposes, DSC analysis was also performed for plain drug FRSD. The sample was kept in aluminum crimped cells and heated for 10 °C min^−1^ between the temperature range of 30°C and 300°C with nitrogen at 40 mL min^−1^. The DSC results obtained for plain drug FRSD, quaternized dendrimers, and their corresponding complexes are shown in Figures 2A–C.

### Dynamic light scattering (DLS) and zeta potential measurements

**FIGURE 3:**
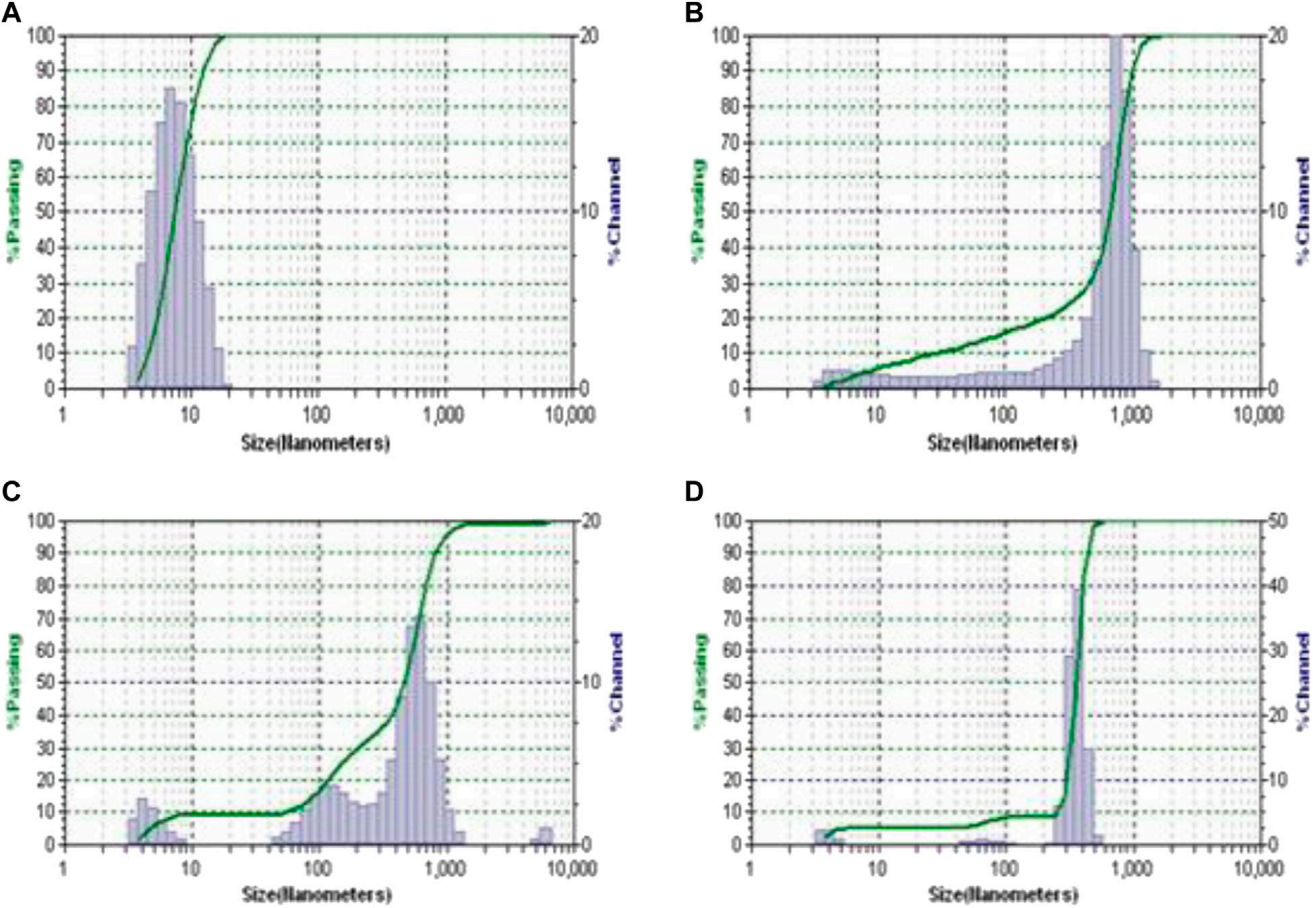
The DLS plots of **(A)** PAMAM (G3), **(B)** PAMAM (G3)-FRSD complex, **(C)** QPAMAM-CH_2_CH_2_OCH_2_CH_2_OH (G3) and **(D)** QPAMAM-CH_2_CH_2_OCH_2_CH_2_OH (G3)-FRSD complex.

In addition to the characterization of complexes through UV-vis and DSC techniques, DLS and zeta potential measurements were also carried out for the selected complex solution QPAMAM-CH_2_CH_2_OCH_2_CH_2_OH (G3) and QPAMAM-CH_2_CH_2_OCH_2_CH_2_OH (G3)-FRSD, along with its parent dendrimer complex PAMAM (G3) and PAMAM (G3)-FRSD, to confirm the interaction between the drug and dendrimer carriers. The sizes of the parent dendrimer, quaternary ammonium drug carrier, and its complex were measured using the complex solution taken from the phase solubility studies with FRSD, and the obtained DLS spectra are shown in Figures 3A–D. In addition, the surface charge of the complex along with their quaternary ammonium dendrimer and parent dendrimer drug carriers were also determined using Zeta potential measurements. The values of the size measured by DLS and surface charges derived from zeta potential were compiled and are shown in Table 2.

**TABLE 2:** The size and zeta potential of the dendrimers and dendrimer-drug complex.

S. No	Dendrimer-drug complex		Size (nm)	Zeta potential (mV)
1	Parent dendrimers (control)	PAMAM (G3)	6.97	7.6
2		PAMAM (G3)-FRSD	63.30	23.7
3	Quaternary ammonium dendrimer drug carriers	QPAMAM-CH_2_CH_2_OCH_2_CH_2_OH (G3)	12.40	11.31
4		QPAMAM-CH_2_CH_2_OCH_2_CH_2_OH-FRSD (G3)	59.80	16.2

^a^ At saturated solutions of FRSD, in 0.35 mM of dendrimer concentration.

### *In vitro* release studies

**FIGURE 4:**
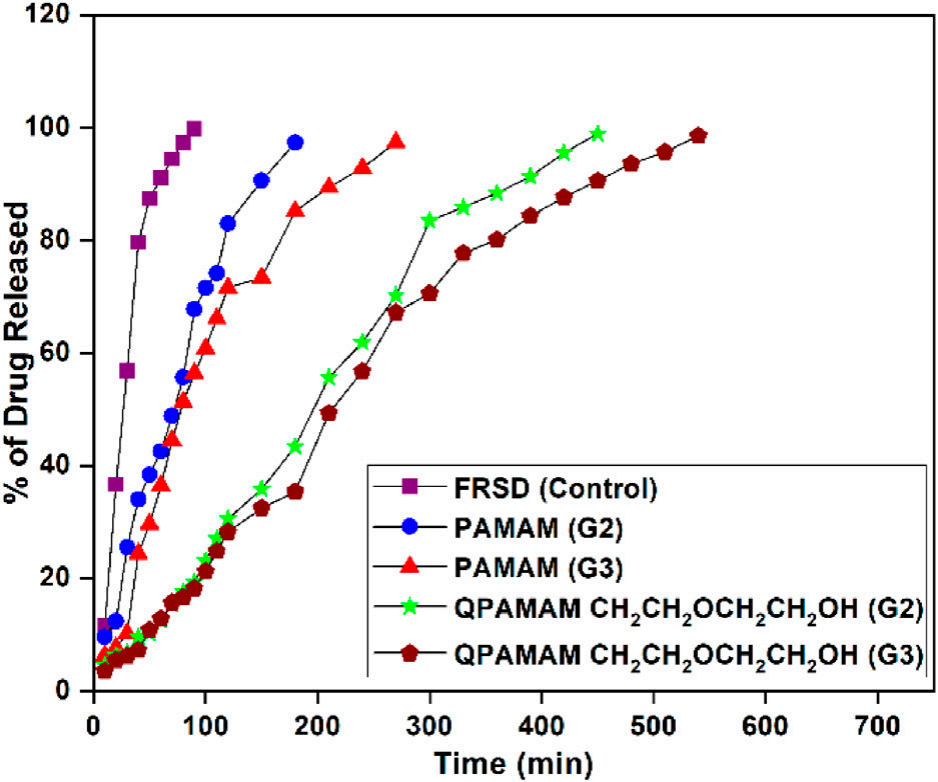
*In vitro* Release behavior of FRSD alone and two types of quaternary ammonium dendrimer drug carriers along with its parent dendrimers.

To examine the efficiency of drug release, newly developed quaternary ammonium dendrimer drug carriers were employed individually for *in vitro* release studies using the representative drug FRSD. The amount of drug released from the respective quaternary ammonium dendrimer carrier-drug formulations QPAMAM-CH_2_CH_2_OCH_2_CH_2_OH (G2) and QPAMAM-CH_2_CH_2_OCH_2_CH_2_OH (G3) was studied in PBS (pH 7.4) *in vitro* by adopting equilibrium dialysis as per the reported procedure (Granero et al., 2010). An aliquot of 5 mL of quaternary ammonium dendrimer carrier-drug formulation solution was taken in a Float-A-Lyzer dialysis tube of 1 kDa MW cutoff to perform an *in vitro* release study. The dialysis tube containing the formulation solution was kept in a glass beaker filled with PBS and stirred to maintain the sink condition. Samples were taken at regular intervals followed by replenishment with an equal amount of fresh PBS. Irrespective of the formulation, the sample withdrawn from the outer phase of dialysis bag was analyzed spectrophotometrically with UV-vis and the absorbance was measured at characteristic λ_max_ of respective drugs. Using the measured absorbance value, the amount of FRSD released from the respective quaternary ammonium dendrimer carrier-drug formulation was calculated. Further, for comparative purposes, *in vitro* drug release from their parent dendrimers PAMAM (G2) and PAMAM (G3), and FRSD drug alone (control), was also carried out. The percentage of drug released from the formulations was calculated using the absorbance value measured at the internal and external medium measured at scheduled intervals, and the results were visualized individually by plotting the percentage of drug released vs. time (Figure 4).

#### Cytotoxicity assay

To establish the suitability for drug delivery application, newly developed quaternary ammonium dendrimer drug carriers should be investigated for cytotoxicity studies under *in vitro* conditions by following the well-established MTT colorimetric assay method. To compare the degree of cytotoxicity of the said quaternary ammonium dendrimer-based drug carriers with their parent dendrimers PAMAM (G2) and PAMAM (G3), a cytotoxicity study was performed using two different cell lines, the Vero cell line (passage numbers 4, 6, and 7) and the HBL-100 cell line (passage numbers 18, 19, and 21), individually. Initially, the two different representative cell lines were incubated individually in MEM (Earle’s salts accompanied with 10% fetal calf serum) with 5% CO_2_ at 37°C, and once the confluence reached 70%–80% these cells were used. To prevent the cell adherence, the respective cells were seeded in 96 well plates and allowed to stand for 48 h. Subsequently, the remaining culture available in each plate was exchanged by adding different concentration of newly prepared the respective quaternary ammonium dendrimer drug carrier as a fresh medium in the range of 3 × 10^−5^ to 1 × 10^−3^ M and allowed to stand for 48 h. Similarly, for comparative purpose, the parental dendrimers PAMAM (G2) and PAMAM (G3) and the control (excluding the dendrimers), were treated with the respective model cells in the medium with the MTT assay. PBS medium was used for incubating respective cells with MTT at a concentration of 1 mg/mL for 3 h. The MTT was reduced to purple formazan in living cells and the formazan generated was dissolved in DMSO. A microplate reader was used to measure the solution absorbance in each well at 570 nm. The absorbance measured with untreated cells (without dendrimer) was set as a positive control and considered as 100% cell viability, and it was directly related to the viability of the cells in each of the wells observed from the treatment of dendrimer dug carriers. The percentage of cell viability for the individual cell lines *versus* the concentration of the respective quaternary ammonium dendrimer drug carrier was plotted along with their parent dendrimers, and the morphological changes observed in the respective cells in the absence and presence of quaternary ammonium dendrimer drug carriers and their corresponding parent dendrimer were photographed. The cell viability plots and photographs are shown in Figure 4.

#### Determination of half maximal inhibitory concentration (IC_50_)

IC_50_ is the acronym for ‘half maximal inhibitory concentration’. The IC_50_ value indicates the concentration of an inhibitor required to inhibit 50% of a biological or biochemical function (i.e. an enzyme, cell, cell receptor, or microorganism). In pharmaceutical research, it is a parameter frequently used to specify the *in vitro* potency of a drug or new chemical entities (NCEs). According to the FDA, *in vitro*, the concentration of drug required for 50% inhibition is IC50, and this value for a particular drug carrier can help the judgment of the permissible/tolerance concentration of that carrier during the preparation of its formulation with a particular drug. In view of this, the IC_50_ values for quaternary ammonium dendrimer drug carriers and their parent dendrimers treated with Vero and HBL-100 cell lines were determined (Table 3).

**TABLE 3:** IC_50_ values of parent and quaternary ammonium dendrimer drug carriers.

Nature of dendrimer carrier	Name of the dendrimer carrier	IC_50_ values in µg/ml			
		Vero cell line		HBL-100 cell line	
		(G2)	(G3)	(G2)	(G3)
Parent dendrimers	PAMAM	50	50	< 25	< 25
Quaternary ammonium dendrimers	QPAMAM-CH_2_CH_2_OCH_2_CH_2_OH	1,000	750	1,000	750

### Stability studies of quaternary ammonium dendrimer-drug formulations

The capability of a pharmaceutical dosage to preserve the chemical, physical, therapeutic, and microbial properties at the time of usage and storage by the patient can be defined as drug stability. Stability studies of each formulation individually were performed both in dark and light conditions at 0°C, 25 ± 2°C, and 60 ± 2°C. Amber-colored vials were chosen for each dendrimer-drug formulation to perform dark condition studies, and colorless vials were used to study the respective formulation under light for a period of 6 weeks in a controlled oven. The stability studies were carried out by aliquotting 5 mL of each formulation into vials. The amber and normal vials were kept at 0°C, room temperature, and in a hot oven for both dark and light conditions for 6 weeks without stirring. Then, the samples from each vial were withdrawn, analyzed, and monitored periodically (every week) for any changes that occurred, including the formation of turbidity, precipitation, crystallization, color, and change in consistency. The observed changes in the form of the above parameters from the first week to the sixth week were expressed in terms of ‘no change’, ‘little change’, ‘considerable change’, and ‘significant change’ (Table 4).

**TABLE 4:** Stability study of two types of quaternary ammonium dendrimer-drug formulation with respect to FRSD.

Dendrimer carrier	Turbidity							Precipitation						Color change						Change in consistency					
	Temperature °C							Temperature °C						Temperature °C						Temperature °C					
	Dark			Light				Dark			Light			Dark			Light			Dark			Light		
	0	RT	At	0	RT	At	0		RT	At	0	RT	At	0	RT	At	0	RT	At	0	RT	At	0	RT	At
1.QPAMAM-CH_2_CH_2_OCH_2_CH_2_OH (G2)	-	-	+	-	+	+	-		+	-	-	+	+++	-	+	+	-	+	+++	-	+	++	-	+	++
2.QPAMAM-CH_2_CH_2_OCH_2_CH_2_OH (G3)	-	-	-	-	+	+	-		-	-	-	+	+	-	-	+	-	+	+	-	-	+	-	-	++

Key: (−) no change; (+) little change; (++) considerable change; (+++) significant change.

## Results and discussion

The FRSD drug molecule has poor oral bioavailability due to its asymmetric transport during absorption (Michael et al., 1974; Granero et al., 2012). Based on this pharmaceutical feature, we developed two types of stable, benign, and efficient drug carrier using dendrimers as a precursor to enhance the solubility and sustained release of FRSD. The main aim of this study was to explore the potential of two types of newly synthesized quaternary ammonium poly (amidoamine) dendrimer drug carrier and thereby establish their suitability for drug delivery applications by conducting representative studies, such as phase solubility, *in vitro* release, and cytotoxicity activity.

### Synthesis and characterization of quaternary ammonium Poly (Amidoamine) dendrimer drug carriers using 2-(2-chloroethoxy)ethanol as a hydroxylating agent

**FIGURE 5:**
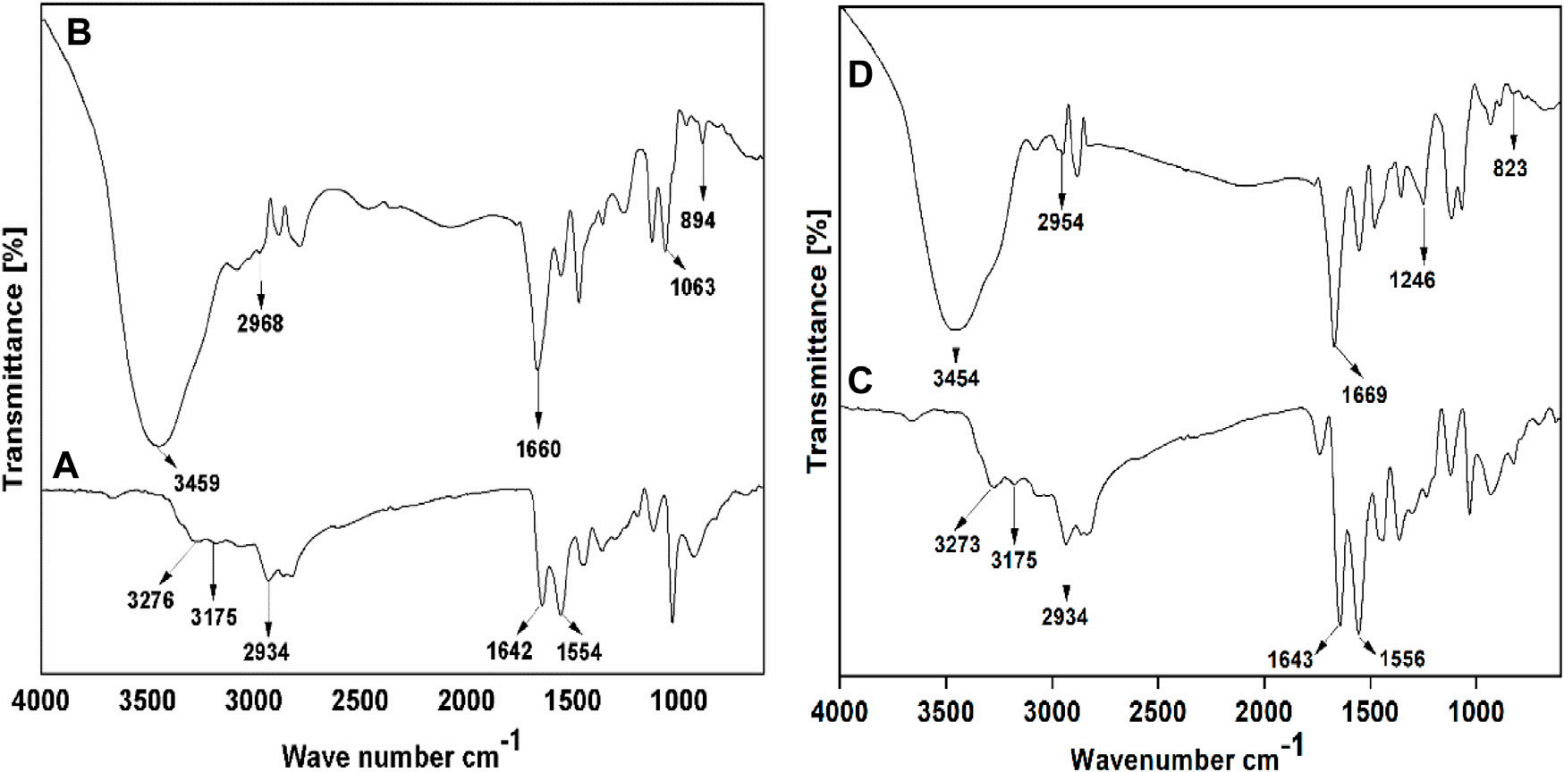
FT-IR spectra of **(A)** PAMAM (G2), **(B)** QPAMAM-CH_2_CH_2_OCH_2_CH_2_OH (G2), **(C)** PAMAM (G3) and **(D)** QPAMAM-CH_2_CH_2_OCH_2_CH_2_OH (G3).

The quaternary ammonium poly (amidoamine) ethoxyethanol dendrimer products were synthesized and characterized using FTIR, ^1^H and ^13^C NMR, and MALDI-TOF spectral techniques. In Figure 5A, the FTIR spectrum of PAMAM (G2) shows characteristic peaks at 3,276 cm^-1^ and 3,175 cm^-1^ due to N-H_str_ of the primary amine, a peak at 2,934 cm^-1^ due to C-H_str_, and peaks at 1,642 cm^-1^ and 1,554 cm^-1^ due to N-H bending. Similarly, the spectrum for PAMAM (G3) in Figure 5C shows characteristic peaks at 3,273 cm^-1^ and 3,175 cm^-1^ due to N-H_str_ of primary amine, a peak at 2,934 cm^-1^ due to C-H_str_, and peaks at 1,643 cm^-1^ and 1,556 cm^-1^ due to N-H bending. The FT-IR spectrum of the synthesized quaternary ammonium poly (amidoamine) ethoxyethanol dendrimer drug carrier QPAMAM-CH_2_CH_2_OCH_2_CH_2_OH (G2) in Figure 5B shows that the peaks caused by N-H_str_ of primary amines disappeared and a new broad peak at 3,459 cm^-1^ appeared due to OH_str_. Additionally, the peaks due to N-H bending of surface amine groups disappeared and a new peak at 1,660 cm^-1^ could be observed. This indicates that the primary amines in PAMAM (G2) are converted to secondary amines, which supports the occurrence of surface hydroxylation. In addition to this, the appearance of an intense peak at 1,063 cm^-1^ corresponded to C-N^+^_str_, indicating the quaternization of the hydroxylated product. Similarly, in Figure 5D, in the FT-IR spectrum of QPAMAM-CH_2_CH_2_OCH_2_CH_2_ OH (G3), the N-H_str_ of primary amines observed in the parent dendrimer disappeared and a new broad peak at 3,454 cm^-1^ appeared due to OH_str_ assigning a hydroxyl group. Additionally, the peaks caused by N-H bending of surface amine groups disappeared and a new peak at 1,669 cm^-1^ could be observed, indicating that the primary amines are converted to secondary amines, thus proving the surface hydroxylation of the former. Furthermore, a new intense peak could be observed at 1,246 cm^-1^, which corresponds to C-N^+^_str_, and this strongly suggests the occurrence of quaternization of the hydroxylated product. To emphasize again, the appearance of OH_str_ and C-N^+^_str_ for both dendrimer drug carriers proves the occurrence of hydroxylation and quaternization on PAMAM (G2) and PAMAM (G3) with 2-(2-chloroethoxy) ethanol and methyl iodide, respectively.

To confirm the occurrence of surface hydroxylation and quaternization on PAMAM (G2) and PAMAM (G3), ^1^H and ^13^C NMR spectral studies were carried out. With ^1^H NMR, QPAMAM-CH_2_CH_2_OCH_2_CH_2_OH (G2) showed an intense signal at δ 3.94 ppm due to OH, a sharp singlet at δ 2.15 ppm due to methyl protons (N-CH_3_), a multiplet signal at δ 3.29 ppm due to methylene protons (N-CH_2_), and an multiplet signal in the range of δ 3.55–3.71 ppm due to methylene protons (–OCH_2_). Similarly, with ^1^H NMR, QPAMAM-CH_2_CH_2_OCH_2_CH_2_OH (G3) showed an intense signal at δ 3.95 ppm due to OH, a sharp singlet at δ 2.15 ppm due to methyl protons (N-CH_3_), multiplet signal at δ 3.28 ppm due to methylene protons (N-CH_2_), and a multiplet signal in the range of δ 3.57–3.76 ppm due to methylene protons (–OCH_2_). For QPAMAM-CH_2_CH_2_OCH_2_CH_2_OH (G2) with 13C NMR, methyl carbons showed peaks at δ 52.21 ppm for N-CH_3_, 60.40 ppm for OH-CH_2_, 69.43 ppm for N-CH_2_, and 71.71 ppm for OH-CH_2_-CH_2_-O. The ^13^C NMR with QPAMAM-CH_2_CH_2_OCH_2_CH_2_OH (G3) showed methyl carbons N-CH_3_ peak at δ 52.18 ppm and methylene carbons peak at 60.40 ppm for OH-CH_2_, 69.45 ppm for N-CH_2_, and 71.77 ppm for OH-CH_2_-CH_2_-O groups. The appearance of the peak caused by alcoholic protons and peaks due to methyl and methylene carbons is strong evidence that surface hydroxylation and quaternization have occurred in both PAMAM (G2) and PAMAM (G3). The structures of dendrimer carriers were further confirmed through their respective MALDI-TOF analysis. The observed spectra for QPAMAM-CH_2_CH_2_OCH_2_CH_2_OH (G2) and QPAMAM-CH_2_CH_2_OCH_2_CH_2_OH (G3) showed the characteristic peaks corresponding to the m/z value at 5,350.64 and 10,514.09, respectively. The obtained experimental m/z values for quaternized dendrimer drug carriers were well in agreement with their corresponding calculated values (5,354 and 10,512).

### Solubility studies of FRSD

It has been widely established that the poor absorption of FRSD was due to its drug solubility, and enhancement of the water solubility of drugs is especially important for their bioavailability (Valentino and Kwame, 2007; Svenson, 2009). To overcome this problem, the two newly developed types of quaternary ammonium dendrimer drug carrier were employed as solubility enhancers for FRSD, and the quantum of solubility was evaluated using the phase solubility technique at seven different concentrations. The degree of solubility was estimated quantitatively using a UV-vis spectrophotometer, and the quantum of drug solubilized was calculated using a calibration curve. A plot was drawn using the concentration of dendrimer against the amount of drug solubilized (Figure 1C), and the calculated quantum of FRSD solubility is shown in Table 1. To ascertain the effect of the surface functional groups in promoting the solubility of poorly soluble drugs, the intrinsic solubility of the drug, 0.0731 mg/ml, was considered as a base value. On comparison, it was inferred that at a maximum concentration, i.e., 0.35 mM, the developed quaternary ammonium poly (amidoamine) dendrimer drug carriers derived from the surface functionalization of the alkyl chain with –OH ether-linked namely, QPAMAM-CH_2_CH_2_OCH_2_CH_2_OH (G2) and QPAMAM-CH_2_CH_2_OCH_2_CH_2_OH (G3), increased the solubility of FRSD 58 times (4.233 mg/ml) and 109 times (8.004 mg/ml), respectively.

The tentative drug molecules bound per molecule of dendrimeric unit were calculated using the directional coefficients of each drug carrier, QPAMAM-CH_2_CH_2_OCH_2_CH_2_OH (G2) and QPAMAM-CH_2_CH_2_OCH_2_CH_2_OH (G3). The directional coefficients were n = 3.28 ± 0.53 and n = 6.49 ± 0.56, respectively, and these values in turn can be interpreted as the number of drug molecules combined/bound by one molecule of dendrimer unit. That is, QPAMAM-CH_2_CH_2_OCH_2_CH_2_OH (G2) and QPAMAM-CH_2_CH_2_OCH_2_CH_2_OH (G3) can bind 3.28 ± 0.53 and 6.49 ± 0.56 drug molecules, respectively, per single molecule of dendrimeric unit. Using the above-mentioned formula, the drug loading efficiencies for QPAMAM-CH_2_CH_2_OCH_2_CH_2_OH (G2) and QPAMAM-CH_2_CH_2_OCH_2_CH_2_OH (G3) were calculated as 64.6% and 58.2%, respectively.

In general, the external groups present in the dendrimer are neutral and contain internal positive binding sites. In brief, the quaternized form containing multicharged cations attracts the anion of the drug and thus FRSD binds electrostatically with QPAMAM-CH_2_CH_2_OCH_2_CH_2_OH (G2)/(G3) in aqueous medium, thus forming the complex, and thereby FRSD becomes internalized within the dendrimer branches. Further, the increased solubility noticed in the presence of the drug carriers, is due to the availability of its (i) enriched surface –OH groups, (ii) the fact it has the lowest number of carbon atoms, and (iii) its sterically free structure due to it having the shortest chain length. It is believed that these three factors contribute effectively and freely to attract the poorly soluble FRSD drug towards the binding sites of the internal quaternary ammonium ions and–OH groups, thus enhancing solubility. Additionally, the comparative solubility values between two generations reveal that the dendrimer carrier derived from higher generation, i.e., PAMAM (G3), increases solubility twofold more than with their corresponding dendrimer drug carriers derived from lower generation PAMAM (G2). Obviously, this is a result of the presence of an–NH_2_ group in PAMAM (G3) and the increased number of surface–OH groups and internal quaternary ammonium ions (or) increased number of interior binding sites.

### Interaction studies of the drug carrier-drug complex

The solubility study confirmed that the complex formed between quaternary ammonium dendrimer drug carriers with drugs is responsible for the solubility enhancement in aqueous medium. To understand the nature of the interaction between drug and quaternary ammonium dendrimer drug carriers, it is necessary to establish the nature of complex formation through thorough characterization.

#### UV-vis spectral studies

The interaction between the drug FRSD and quaternary ammonium dendrimer drug carriers was analyzed using a UV-vis spectrophotometer with the complex forming solution, and the obtained spectra are shown in Figures 1A, B. A red shift was observed in the quaternary ammonium dendrimer drug carrier-drug complex, indicating an interaction between the drug carrier and drug with λ_max_ at 277 nm compared with λ_max_ at 271 nm with the control. In general, any shifts noted in UV absorption maxima are indicative of complex formation, confirming the presence of interaction between components (Buhleier et al., 1978). In this study, the electrostatic force of interaction between the cationic groups of quaternary ammonium dendrimer drug carriers and the anionic group of FRSD could be responsible for this red shift. The formation of the red shift for the complex solutions at corresponding λ_max_ directly supports the establishment of a complex between quaternary ammonium dendrimer drug carriers and FRSD through electrostatic interaction.

#### Differential scanning calorimetry (DSC) analysis

The interaction between quaternary ammonium dendrimer drug carriers and FRSD complex solution was also established by DSC analysis. The selected complex solution with a higher generation of quaternary ammonium dendrimer drug carrier, i.e., QPAMAM-CH_2_CH_2_OCH_2_CH_2_OH (G3) and its complex QPAMAM-CH_2_CH_2_OCH_2_CH_2_OH (G3)-FRSD, at a concentration of 0.35 mM, was lyophilized and the obtained solid complex was analyzed through DSC analysis along with its control drug, FRSD (Figures 2A–C). The DSC thermogram of FRSD in Figure 2A shows its characteristic sharp endothermic peak at 228.76 °C, which is associated with the melting point of the drug and indicates its crystalline nature, and its degradation product shows an endothermic peak at 272.94 °C (Murugan et al., 2015). As the synthesized dendrimer drug carriers were highly hygroscopic, the DSC showed a broad endothermic peak at 124.0°C for QPAMAM-CH_2_CH_2_OCH_2_CH_2_OH (G3) (Figure 2B), corresponding to its dehydration. On the contrary, the thermogram for the complexes shows only the peak of its quaternary ammonium dendrimers, and the peak caused by the drug has almost disappeared. The complex solution shows an endothermic peak for the dendrimer drug carrier alone, i.e., 143.42 °C for QPAMAM-CH_2_CH_2_OCH_2_CH_2_OH (G3)-FRSD (Figure 3C). In other words, the complex of the quaternary ammonium dendrimer drug carrier does not show a peak that is caused by the drug. This complete absence of an FRSD peak suggests that FRSD is in amorphous state or residing inside the modified dendrimers. Additionally, the disappearance of endothermic peaks associated with FRSD is an indication of the change in its crystalline structure in dendrimer solutions (Cheng et al., 2008a). Similar observations were observed in our previous study (Patel et al., 2008), and it has also been suggested that the disappearance of a drug endothermic peak in a particular DSC thermogram of the respective complex is strong evidence of interactions taking place between the carrier and the drug (Prahlad and Rajendrakumar, 2003; Panda et al., 2011). Therefore, we conclude that FRSD complexed with the quaternary ammonium dendrimer drug carriers.

### Dynamic light scattering (DLS) and zeta potential measurement

Further, to examine the presence of an interaction between the dendrimer drug carrier and the drug, DLS and zeta potential measurements were also carried out using the complex solution obtained from solubility studies. The size of the dendrimers and their corresponding complexes were measured, and the plots are shown in Figure 2 a-d. The size of PAMAM (G3) was determined as 6.97 nm, whereas the size of its complex PAMAM (G3)-FRSD was 63.30 nm. The size of the quaternary ammonium dendrimer drug carrier QPAMAM-CH_2_CH_2_OCH_2_CH_2_OH (G3) was 12.40 nm, and its complex, QPAMAM-CH_2_CH_2_OCH_2_CH_2_OH (G3)-FRSD, was 59.80 nm. A large increase in the size of the PAMAM (G3)-FRSD compared to QPAMAM-CH_2_CH_2_OCH_2_CH_2_OH (G3)-FRSD was observed, thus confirming attachment of the anionic FRSD drug on the surface of the –NH_2_ groups. By contrast, the size difference of its complex was minimal, suggesting that the FRSD molecule may be internalized inside the branch point of the quaternary ammonium ions and thereby formed a complex. These observations are similar to earlier results (Murugan et al., 2015) that showed that the association of plasmid DNA molecules with QPAMAM-OH (G4) dendrimer results in the formation of polyplexes for gene delivery.

The surface charges of the dendrimer drug carriers and their corresponding complex were also measured using zeta potential (Table 3). The zeta potential is an indicator for the charge present on the surface of particles, which provides evidence for the stability of formulations and interactions with cellular membranes. The observed zeta potential for PAMAM (G3) and QPAMAM-CH_2_CH_2_OCH_2_CH_2_OH (G3) and its complex PAMAM (G3)-FRSD and QPAMAM-CH_2_CH_2_OCH_2_CH_2_OH (G3)-FRSD was 7.6 mV and 11.31 mV and 23.7 mV and 16.2 mV, respectively. The observed charge indicates that the cationic surface is neutralized by the anionic carboxyl group of FRSD. The observed variation in size and charge with DLS and zeta potential measurements supports the evidence for the interaction between quaternary ammonium dendrimer drug carriers and FRSD.

#### *In Vitro* release studies

The very purpose of developing a new drug carrier is to deliver that particular drug to the targeted site for action in a controlled/sustained manner, thus having potential medical applications. The possibilities of overdosing and side effects can be reduced by avoiding the therapeutic concentration fluctuations of drugs in the body. (Langer, 1998). Previous reports showed that poorly soluble/hydrophobic drugs can encapsulate into PAMAM- and PPI-derived dendrimer carriers within their hydrophobic unimolecular micellar structure, and the formulations obtained from these carriers are suitable for drug delivery *via* different administration routes (Langer, 1998; Prahlad and Rajendrakumar, 2003; Cheng et al., 2008a; Patel et al., 2008; Wu et al., 2009; Hu J. et al., 2010; Panda et al., 2011). The biocompatibility of drugs loaded in these dendrimer carriers can be developed for sustained release by reducing the dose schedule and improving patient compliance (Gupta et al., 2007; Cheng et al., 2008b). To prove the releasing ability of newly developed drug carriers, an *in vitro* release studies were carried out using quaternary ammonium poly(amidoamine) dendrimer drug carrier-drug formulations, their corresponding parent formulations, and FRSD as a control, and the results were compared. *In vitro* release behavior was studied using the equilibrium dialysis method and a drug release plot of the percentage of drug released *versus* time was drawn and is shown in Figure 3. From the plots, the quantum of time required to release the maximum percentage of drug, i.e. 95%, from each formulation was determined and compared. Comparative study of these plots shows that within 90 min, almost 99% of FRSD is released from its control formulation and during the same 90 min, PAMAM (G2)-FRSD and PAMAM (G3)-FRSD are released up to 66% and 67%. The newly developed drug carriers, QPAMAM- CH_2_CH_2_OCH_2_CH_2_OH (G2)-FRSD and QPAMAM-CH_2_CH_2_OCH_2_ CH_2_OH (G3)-, released only 19 and 15% FRSD, respectively for 90 min. The time taken for 95% of drug release from PAMAM (G2) and PAMAM (G3) carriers was 180 and 270 min, whereas from the developed quaternary ammonium dendrimer drug carrier it was 420 min and 510 min, respectively. The delay in release time is strong evidence for sustained drug release.

In general, it is known that the sustained release of a drug by any drug carrier is possible, provided the complex formed between them is stable due to the interaction or the effective internalization of the drug within the drug carrier. In the absence of a carrier or in the presence of medium alone, FRSD was quickly released up to the maximum percentage (95%) over 90 min. The time required to release the same percentage of drug was relatively extended from 90 min to 150 min in the presence of commercial PAMAM (G2) dendrimer carrier and was extended to 180 min for commercial PAMAM (G3) dendrimer carrier. The PAMAM (G3) carrier showed relatively better delayed release than PAMAM (G2). It is due to availability of a two-fold number of quaternary ammonium groups, that interact with much drug molecules and release them slowly. The formulation obtained from the quaternary ammonium dendrimer drug carrier with functionalization of ether-linked–OH 2-[2-(2-chloroethoxy) ethoxy]ethanol, namely QPAMAM-CH_2_CH_2_OCH_2_CH_2_OH, showed better sustained release of the drug than with parent dendrimers. Initially, the drug molecule attached on the surface moiety is released and then the drug encapsulated in the internal cavities of the carrier is released slowly because it should cross barriers, such as long chain length as well as the crowded surface groups, resulting in sustained drug release. The superior sustained release behavior observed in the presence of carrier is due to 1) the availability of more surface–OH groups, 2) more carbon atoms, and 3) the sterically hindered structure due to carbon chain length. These three factors co-operatively function to enable the sustained release of FRSD.

The complexes formed between the quaternary ammonium dendrimer carrier and the drug mainly occurs *via* electrostatic interaction, which depends on the ionic strength of the medium. The formation of a stable complex between them in aqueous medium supports it. In other words, the newly developed quaternary ammonium dendrimer drug carriers with increased hydrophobic character and high steric hindrance show sustained drug release due to the formation of stable complexes in the form of HCl (A^−^)_n_ (QPAMAM-X (G2)/(G3)^n+^ (where A is FRSD and X represents different types of dendrimer drug carriers). The formation of this stable complex greatly contributes to the sustained release of the drug.

#### Cytotoxicity assay

**FIGURE 6:**
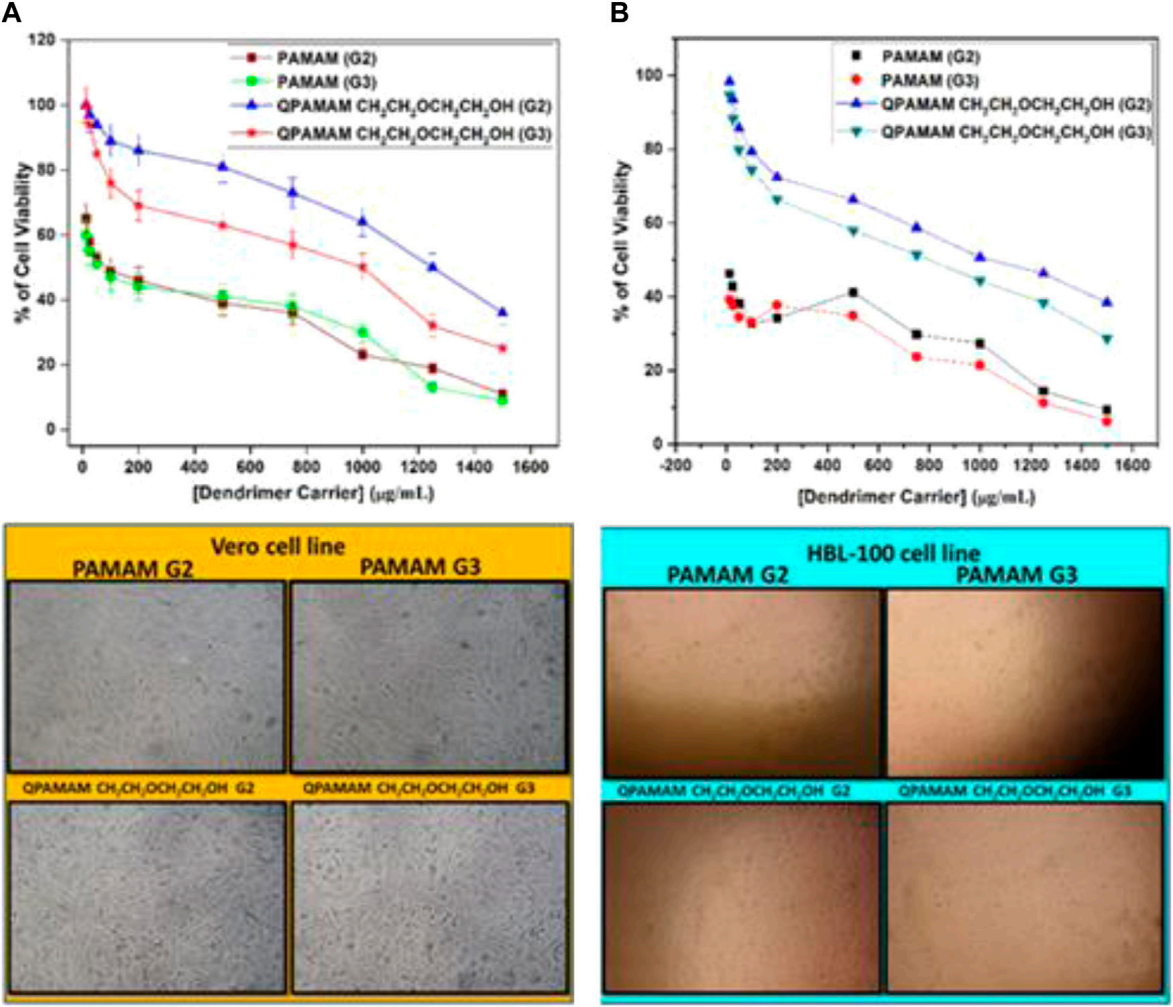
Cytotoxicity of parent dendrimers and quaternary ammonium dendrimer drug carriers at different concentrations on treated with **(A)** Vero cell line, **(B)** HBL 100 cell line and its Photographs.

Overall, the biocompatible polymeric drug carrier should be obtained in a toxin-free form (Buhleier et al., 1978). To prove the non-toxicity of our newly developed quaternary ammonium dendrimer drug carriers, cytotoxicity studies were performed under *in vitro* conditions using the well-established MTT colorimetric assay. MTT enters the cells and locates to the mitochondria, which are reduced to an insoluble dark purple-colored formazan product. The cells with MTT are then solubilized with the organic solvent DMSO, and the solubilized formazan reagent is estimated spectrophotometrically. As the reduction of MTT can only occur in metabolically active/living cells, the level of activity is a measure of the viability of the cells, and the total mitochondrial activity of most cell populations is related to the number of viable cells. The morphological changes observed in the respective cell lines in the absence (control cells) and presence of newly developed quaternary ammonium dendrimer drug carriers and the corresponding parent dendrimers were photographed and are shown in Figure 6. The control cells of the Vero cell and HBL-100 cell lines clearly show the appearance of cell viability and confluence growth of the cells. By contrast, when the same cells were treated with the parent dendrimers, PAMAM (G2) and PAMAM (G3), the shape of the cells changed, resulting in a drastic decrease in cell intensity. The drug carriers QPAMAM-CH_2_CH_2_OCH_2_CH_2_OH (G2) and QPAMAM-CH_2_CH_2_OCH_2_CH_2_OH (G3) upon treatment with Vero cells (Figure 6A) exhibited much higher cell viability (97% and 94%, respectively). The cell viabilities for PAMAM (G2) and PAMAM (G3) were 60% and 55% at the same concentration. Upon treatment with the HBL-100 cell line, QPAMAM--CH_2_CH_2_OCH_2_CH_2_OH (G2) and QPAMAM-CH_2_CH_2_OCH_2_CH_2_OH (G3) had cell viabilities of 98% and 94%, respectively (Figure 6B), and the cell viability of PAMAM (G2) and PAMAM (G3) was 42% and 37%. The plots in Figure 6B show that the newly developed dendrimer drug carriers are less cytotoxic than their native dendrimers. These results show that the developed drug carriers will increase the tolerance concentration effectively at the time of their administration. Additionally, they reveal that the developed dendrimer carriers are better at delivering FRSD, resulting in reduced cytotoxicity and enhanced biocompatibility. The drug carrier with the cationic surface group must be neutralized to reduce cytotoxicity. In fact, an earlier study by Devarkonda et al. (2007) used unmodified dendrimers as drug carriers with no toxicology analysis. In this study, the developed drug carriers QPAMAM-CH_2_CH_2_OCH_2_CH_2_OH (G2) and QPAMAM-CH_2_CH_2_OCH_2_CH_2_OH (G3), with a neutral surface group, showed reduced cytotoxicity with increased biocompatibility upon interaction with these cell lines compared with their native dendrimers.

#### Determination of half maximal inhibitory concentration (IC_50_)

The determination of IC_50_ helps tolerance concentration estimation of a particular carrier during the preparation of a formulation. IC_50_ values were determined for quaternary ammonium dendrimer-based drug carriers and their corresponding parent dendrimers. The IC_50_ values were determined from the respective cell viability plots obtained by plotting the percentage of cell viability against dendrimer concentration. IC_50_ values for each cell line were determined by observing the concentration of a dendrimer carrier at which 50% of the total cells remained viable (Table 3). IC_50_ values for the Vero cell line for parent dendrimers PAMAM (G2) and PAMAM (G3) were 50 μg/mL, whereas for HBL-100 cell lines it was less than 25 μg/mL for both PAMAM (G2) and PAMAM (G3) dendrimers. By contrast, the IC_50_ values for the quaternary ammonium dendrimers were higher than their corresponding parent dendrimers. These results show that tolerance concentration can be increased by quaternary ammonium dendrimer drug carriers during their formulation and subsequent administration. The observed order found in the IC_50_ values can be justified by the argument that any reduction in cell viability is in turn related to the IC_50_ values, mainly because of the negative charge of the neutral surface group screening effect on the cell membranes. This is directly reflected in the observed order of IC_50_ values.

### Stability studies of quaternary ammonium dendrimer-drug formulations

It has been proven that newly developed quaternary ammonium poly (amidoamine) dendrimer drug carriers are effective at 1) solubilizing the poorly soluble drug, 2) sustaining *in vitro* release, and 3) reducing cytotoxicity. However, for the application of these drug carriers in real-time drug delivery, it is essential to establish and prove the stability of the developed dendrimer carrier-drug formulations for better storage. As explained in Materials and Methods, the stability assessment of the formulations was performed at different temperatures (0°C, 25°C ± 2°C and 60°C ± 2°C) both in dark and light, without stirring, for 6 weeks. The samples from each formulation were withdrawn and analyzed periodically (every week) for up to 6 weeks and examined for the appearance of any turbidity, precipitation, crystallization, change in color, and change in consistency. The observed changes after 6 weeks were labeled as either ‘no change’, ‘little change’, ‘considerable change’, or ‘significant change’. Table 4 indicates that the newly developed quaternary ammonium dendrimer carrier-drug formulations are sufficiently stable under the experimental conditions specified above. All formulations used in this analysis were quite stable even at 60 ± 2°C, under dark (amber-colored vials) conditions. However, a slight change in color, as well as precipitation, was observed after a period of 6 weeks when kept in the light (colorless/transparent vials). Further, the dark condition results suggested that no significant changes occurred irrespective of formulation and hence the newly developed dendrimer-based formulations were proven to be stable even at elevated temperatures. Therefore, considering parameters, such as (i) change in turbidity, (ii) precipitation, (iii) change in color, and (iv) change in consistency for stability studies, all dendrimer carrier-drug formulations are stable, even after 6 weeks. Overall, it can be concluded that the formulations should be stored at low temperatures in the dark.

## Conclusion

Dendrimers are ideal carriers in drug delivery applications due to their tailor-made options with regard to the architecture of the dendrimer surface. In this study, two types of PAMAM dendrimer-based drug carriers, QPAMAM-CH_2_CH_2_OCH_2_CH_2_OH (G2 and G3), were synthesized, characterized, and examined as drug carriers for the poorly soluble drug FRSD. They achieved enhanced drug solubilization due to their drug loading potential, and the drug solubility was promoted to class III from class IV as per BCS norms. Additionally, *in vitro* drug release proceeded in a sustained manner. This solubility enhancement and sustained release is facilitated by complex formation through interaction between QPAMAM-CH_2_CH_2_OCH_2_CH_2_OH and FRSD. The increased cell viability observed with the Vero and HBL 100 cell lines, with their reduced cytotoxicity compared with native dendrimers, leads to high biocompatibility. During the dendrimer-drug formulation, the tolerance concentration increased effectively due to the IC_50_ value. The studies showed that dendrimer carrier-drug formulations were stable for more than 6 weeks; therefore, it can be concluded that the formulations can be stored at low temperatures in the dark. The modification of surface amine groups in PAMAM dendrimers and internal quaternization are shown to play a critical role in the increased solubility, sustained drug release, and reduced cytotoxicity of the dendrimer-drug complex. Hence, the developed quaternary ammonium dendrimer drug carrier could provide new routes for the development of novel dendrimer drug delivery platforms in the future. Therefore, the present drug delivery methodology will become an integral part of medicine in the future.

## Data Availability

The original contributions presented in the study are included in the article/supplementary material, further inquiries can be directed to the corresponding author.
