# Erratum: Development of a quaternary ammonium poly (amidoamine) dendrimer-based drug carrier for the solubility enhancement and sustained release of furosemide

**DOI:** 10.3389/fchem.2023.1236207

**Published:** 2023-06-13

**Authors:** Frontiers Production Office

**Affiliations:** Frontiers Media SA, Lausanne, Switzerland

**Keywords:** PAMAM dendrimer, congestive heart failure, solubility enhancement, sustained release, biocompatibility

Due to an Editorial Office error, the following declaration was missed from the **Conflict of Interest** statement: “The handling editor NJ declared a past collaboration with the author EM.”

The publisher apologizes for this mistake. The original version of this article has been updated.

